# Hysterectomy in Puerperal Sepsis1Read before the Buffalo Academy of Medicine.

**Published:** 1896-05

**Authors:** Herman E. Hayd

**Affiliations:** 78 Niagara Street


### HYSTERECTOMY IN PUERPERAL SEPSIS.[1]

HERMAN E. HAYD, M.D., M.R.C.S., Eng.

THE subject assigned me for discussion this evening resolves itself into a very much-debated question in surgery: Is hysterectomy ever justifiable in puerperal sepsis? And on account of the gravity of this surgical procedure, together with the difficulties associated with absolute diagnosis, there will always be room for honest doubt and spirited controversy.

It has been shown by the previous speakers that puerperal infection readily takes place through rents in the vagina and cervix, from the decomposition and breaking down of retained clots and secundines, from adherent portions of placental tissue and from septic endometritis and metritis, either due to these enumerated

1. Read before the Buffalo Academy of Medicine.

causes or independent of them. It has also been satisfactorily demonstrated that septic peritonitis and post-puerperal fever may result from a latent pyo-salpingitis or tubo-ovarian abscess, from a suppurating ovarian cystoma, from an intraligamentary cyst or fibroid, or from dermoid or pelvic cellulitis, due to lymphatic absorption and infection consequent upon injuries of the cervix and uterus during parturition or operation.

Many cases have been operated upon successfully and life has been frequently saved by removing these different, recognized, pathological conditions; in fact, such complications every modern surgeon would not hesitate to operate for at once.

There exists, however, a certain group of cases where the tubes and ovaries are but slightly involved, if at all, and where the uterus, although movable, is still the seat of grave lesions, say a septic endometritis and metritis, with pus and multiple abscesses in the walls of the organ. It is in this definite, but limited, class where the most delicate and discriminating judgment is required to make a diagnosis, but when it can be made, there can be no question about the operative indications. Pus here, as elsewhere, must be evacuated. It would, however, be folly to expect that anything short of total extirpation could relieve such a condition; therefore the whole septic uterus must be taken away and the patient given at least the only chance to save life.

Dr. Baldy, of Philadelphia, has written very largely on this subject and has collected a number of cases from his own work and that of Kelly, Hirsh, Smith, Davis and others.

Hysterectomy in a puerperal patient suffering from double pyo-salpinx of gonorrheal origin and recovery is not an uncommon result; and perhaps it may be laid down as a broad proposition that bilateral disease of the annexa in a puerperal patient should demand total extirpation of the uterus and its appendages; because we can never be positive that there does not exist in the uterus septic foci, and accumulations of pus within its muscular layers. Moreover, the time required to remove the uterus and the shock and dangers of the radical operation are but so slightly increased that most prominent operators advocate the most extreme measures.

In a paper written by Baldy, in which he reports fifteen operations, I find in six of the cases there was no disease of the appendages, and the lesions in the uterus are stated to have been pus in the uterine walls and uterine sinuses—disintegration of the uterine walls and gangrene; and yet four of these patients recovered. Of the remaining nine, tubal, ovarian and broad ligament infiltration and suppuration existed in four, and two of these recovered by operation. There can be no question, then, that these cases, representing as they do the work of honest and conscientious observers, justifies the operation of hysterectomy for puerperal sepsis, either with or without tubal or broad ligament involvement.

Notwithstanding the more careful instruction in obstetrics and a better appreciation of the more common causes of puerperal infection, a great many women are every day dying from the effects of puerperal septicemia, and the more we can impress ourselves with the local origin of this horrible disease the sooner will we find the means to combat and prevent it. It seems to me that this disease—so-called puerperal fever—is of local origin, and its treatment must very largely be topical. Refinements in diagnosis and more perfect methods of examination will from time to time be developed, and then even the acute fulminating varieties will be operated upon early and, perhaps, life saved. The great surgical axiom must be, operate early, before too great blood infection and dyscrasia has taken place. Of course, in assuming such advantages, terrible responsibilities must inevitably follow, and so great will be the dangers of too early and too frequent operations that this life-saving, scientific procedure will be much abused and many deaths result from its careless application.

It must not be forgotten that most cases of puerperal infection get well by simple sedative treatment, endometritis, metritis, salpingitis, ovaritis and peritonitis—complications frequently seen—undergo resolution and tubes previously closed and adherent become free and patulous.

But this is more often the case when the inflammatory exudates do not go on to suppuration, and I am sure I am justified in making the statement, when pus can be diagnosticated, tentative, tonic and building-up treatments are out of the question, and an incision, whether by laparatomy or vaginal hysterectomy, or simply an incision through the vaginal vault, is the only course to pursue, and it should be done with the greatest dispatch.

Some men, and particularly Mordecai Price, argue that hysterectomy for puerperal septicemia is a criminal operation, and maintain that all cases of puerperal sepsis which can be influenced at all will be relieved by the finger and intrauterine douche. There can be no doubt that many cases of marked infection have been cured by these simple measures, and if we add to them the curette and gauze packing in cases where septic endometritis and metritis exists, the group grows very much larger ; but there will always remain a limited number of desperate cases where pus exists in the muscular wall and uterine sinuses and nothing short of total hysterectomy promises anything. This class of cases I have already alluded to in this paper, and after studying very carefully those reported by Baldy, I am convinced that surgery offers some hope when the diagnosis can be established. It is, therefore, a reasonable expectation in a given case of pronounced sepsis where the uterus has been carefully and thoroughly cleaned out, and where chills, rapid pulse and leaky skin continue, to hope for good results by an early hysterectomy. Delay in such a case is cowardice and robs us of what little hope of success there remains.

Occasionally epidemics of puerperal fever occur, where the systemic infection is so overwhelming and the local evidences of the disease so slight and death so certain and rapid, that surgery offers but very little, if any, assistance.

78 Niagara Street.

